# 8-Hy­droxy-6-meth­oxy-7-(3-methyl­but-2-en­yloxy)coumarin (capensine)

**DOI:** 10.1107/S241431462100451X

**Published:** 2021-05-11

**Authors:** Azimjon Mamadrakhimov, Luqmonjon Mutalliyev, Sirojiddin Abdullaev, Khamid Khodjaniyazov, Lidiya Izotova, Haji Akber Aisa

**Affiliations:** a Institute of Bioorganic Chemistry, UzAS, M. Ulugbek Str. 83, 100125, Tashkent, Uzbekistan; b National University of Uzbekistan, University Str. 4, Tashkent 100174, Uzbekistan; c Key Laboratory of Plants Resources and Chemistry of Arid Zone, Xinjiang Technical Institute of Physics and Chemistry, Chinese Academy of Science, Urumqi 830011, People’s Republic of China; Purdue University, USA

**Keywords:** crystal structure, coumarin, capensin, isolation, natural product, hydrogen bonding

## Abstract

The title coumarin derivative, C_15_H_16_O_5_, was isolated from the roots of *Sophora japonica*. The coumarin (2*H*-chromen-2-one) fragment is almost planar, with an r.m.s. deviation of 0.0356 Å. The carbon atom of the meth­oxy substituent is coplanar with the benzo­pyran oxa-heterocycle. The 3-methyl­but-2-en­yloxy group is disordered over two sets of sites with occupation factors of 0.920 (3) and 0.080 (3). In the crystal, mol­ecules are linked by O—H⋯O and C—H⋯O hydrogen bonds into chains propagating along the [101] direction.

## Abstract 2

The title coumarin derivative was isolated from the plants of *Sophora japonica*. In the crystal, mol­ecules are linked by O—H⋯O and C—H⋯O hydrogen bonds into chains along the [101] direction.

## Graphical abstract

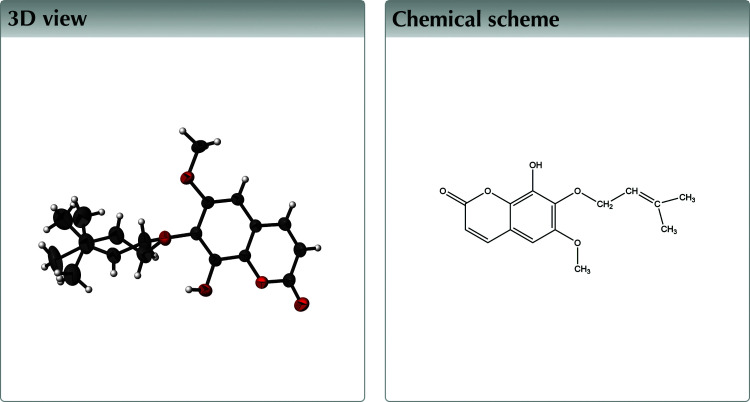

## Structure description

Coumarin derivatives constitute the core structure of various natural products and are a pharmacophore of numerous medicinal agents with anti­microbial, anti­fungal or anti­oxidant properties (Hulushe *et al.*, 2020 ▸; Mladenović *et al.*, 2009 ▸; Al-Ayed, 2011 ▸). The properties of coumarin derivatives are also of inter­est as targets for synthetic organic chemists and serve as inter­mediates in the synthesis of new biologically active compounds. In addition, certain derivatives of coumarins are known to induce apoptosis by cytochrome C release and caspase activation (Johansson *et al.*, 2003 ▸). A number of articles report coumarin derivative such as 7-hy­droxy-coumarin (Gourdeau *et al.*, 2004 ▸), 7,8-diacet­oxy-4-methyl­coumarin or 7,8-diacet­oxy-4-methyl-coumarin (Skommer *et al.*, 2006 ▸; Patchett *et al.*, 2000 ▸) with selective cytotoxicity towards cancer cells, which inhibit the growth of certain types of lung cancer cells.

**Figure 1:**
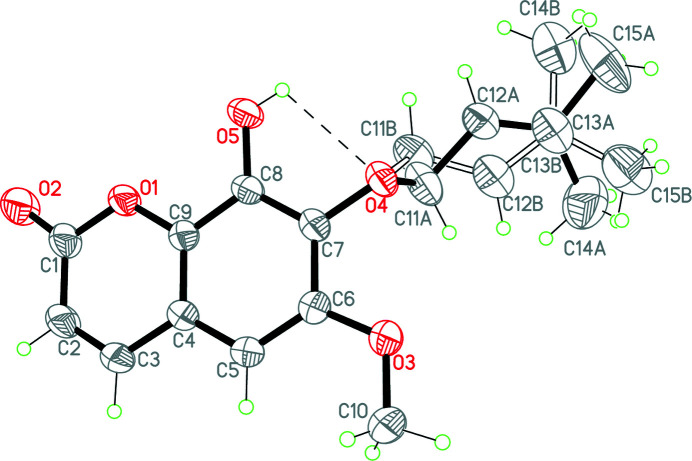
The mol­ecular structure of the title compound with atom labelling. Displacement ellipsoids are drawn at the 50% probability level.

The title compound, the coumarin capensine, was first isolated from *Haplofyllum obtusifolium* and its atomic connectivity has been established by chemical and spectroscopic methods (Matkarimov *et al.*, 1980 ▸; Vdovin *et al.*, 1987 ▸). The same coumarin was isolated from the roots of *Sophora japonica*. Slow evaporation from a solution in methanol yielded monoclinic crystals with space group *P*2_1_/*n* with one crystallographically independent mol­ecule. The mol­ecular structure of the title compound is presented in Fig. 1 ▸. The benzo­pyran ring system is practically planar, the r.m.s. deviation from planarity being 0.0356 Å. The meth­oxy substituent at atom C8 lies almost within the plane of the benzo­pyran oxa-heterocycle. The torsion angle C7—C6—O3—C10 is 178.18 (3). The 3-methyl­but-2-en­yloxy substituent at atom C7 is disordered over two sets of sites by a rotation around the C11—C12 bond. The two orientations are not equivalent – the site occupation factors are 0.920 (3) and 0.080 (3).

**Figure 2:**
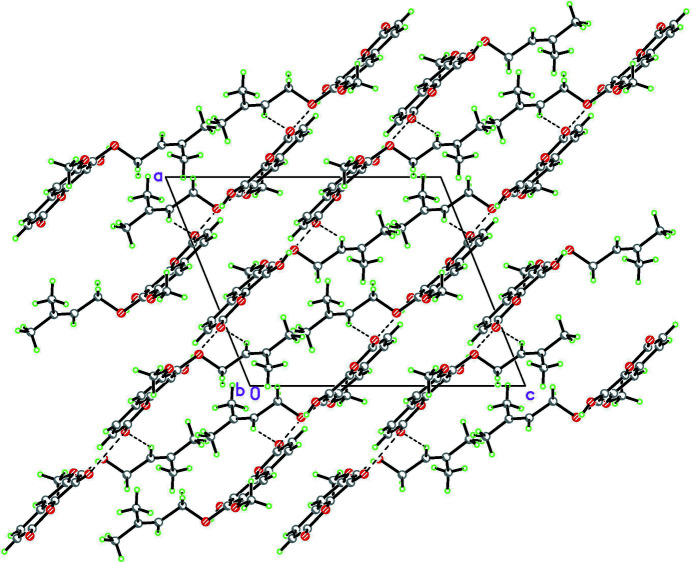
Crystal structure of the title compound in a projection on the (101) plane. Inter­molecular hydrogen bonds are shown as dashed lines. The figure shows only the major occupancy component of the disordered 3-methyl­but-2-en­yloxy substituent at atom C7.

The hydroxyl group O5—H at C8 participates in a bifurcated hydrogen bond: intra­molecular and inter­molecular (Table 1 ▸). The intra­molecular hydrogen bond O5—H5⋯O4 [2.758 (1) Å, 111°] closes a five-membered ring with an *S*(5) graph-set motif (Etter, 1990 ▸). The same hydroxyl H atom also bonds towards the ester keto oxygen atom O2 in a neighboring mol­ecule (at − 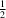 + *x*, 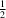 − *y*, − 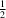 + *z*), which, in turn, is hydrogen-bonded to the C11*B* (C12*A*) atoms of the 3-methyl­but-2-en­yloxy substituent at atom C7 of the first mol­ecule *via* C11*B*—H11*D*⋯O2^i^ and C12*A*—H12*A*⋯O2^i^ hydrogen bonds (Table 1 ▸), thus connecting mol­ecules into chains propagating along the [101] direction (Fig. 2 ▸).

## Synthesis and crystallization

The title compound was isolated from the roots of *Sophora japonica*. The roots (2.5 kg) of *S. japonica* were extracted with ethanol at room temperature, which afforded a light-yellow residue (228.1 g) after solvent evaporation under reduced pressure. The residue was diluted with water (1:1), washed with non-polar solvents (hexane, petroleum ether, gasoline) to remove lipophilic substances, and then subjected to sequential liquid–liquid extraction with chloro­form, ethyl acetate, and *n*-butanol. The obtained chloro­form fraction (30.4 g) was subjected to column chromatography on silica gel in gradient solvent systems; coumarins were isolated from the eluates obtained by repeated chromatography on a polyamide sorbent, preparative TLC on Silufol UV-254 in the following system: chloro­form–petroleum ether–ethanol (8:2:2), *R*_f_ = 0.74 and fractional crystallization from chloro­form. The yield of capensine was 55 mg (0.0022%), m.p. 139–141°C. Suitable crystals for X-ray structural analysis were obtained by slow evaporation from a solution in methanol at room temperature.

## Refinement

Crystal data, data collection and structure refinement details are summarized in Table 2 ▸. Disorder was observed for the 7-(3-methyl­but-2-en­yloxy)group. The disordered atoms C11–C15 were modelled over two positions. The geometries of the two moieties were restrained to be similar to each other (SAME command of *SHELXL*, e.s.d. used was 0.02 Å). *U^ij^* components of disordered atoms were restrained to be similar for atoms closer to each other than 2.0 Å (SIMU restraint of *SHELXL*, e.s.d. used was 0.01 Å^2^). The occupancy ratio refined to 0.920 (3):0.080 (3).

## Supplementary Material

Crystal structure: contains datablock(s) I. DOI: 10.1107/S241431462100451X/zl4043sup1.cif

Structure factors: contains datablock(s) I. DOI: 10.1107/S241431462100451X/zl4043Isup2.hkl

CCDC reference: 2080647

Additional supporting information:  crystallographic information; 3D view; checkCIF report

## Figures and Tables

**Table 1:** Hydrogen-bond geometry (Å, °)

*D*—H⋯*A*	*D*—H	H⋯*A*	*D*⋯*A*	*D*—H⋯*A*
O5—H5*A*⋯O4	0.82	2.35	2.7580 (13)	111
O5—H5*A*⋯O2^i^	0.82	2.09	2.8484 (13)	153
C11*B*—H11*D*⋯O2^i^	0.97	2.41	3.19 (3)	137
C12*A*—H12*A*⋯O2^i^	0.93	2.54	3.3468 (19)	145

Symmetry code: (i) 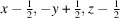 .

**Table 2:** Experimental details

Crystal data	
Chemical formula	C_15_H_16_O_5_
*M*_r_	276.28
Crystal system, space group	Monoclinic, *P*2_1_/*n*
Temperature (K)	293
*a*, *b*, *c* (Å)	11.4373 (2), 9.2045 (1), 13.9862 (2)
β (°)	112.030 (2)
*V* (Å^3^)	1364.89 (4)
*Z*	4
Radiation type	Cu *K*α
μ (mm^−1^)	0.84
Crystal size (mm)	0.30 × 0.25 × 0.20

Data collection	
Diffractometer	Rigaku Oxford Diffraction Xcalibur, Ruby
Absorption correction	Multi-scan (*CrysAlis PRO*; Rigaku OD, 2020 ▸)
*T*_min_, *T*_max_	0.707, 1.000
No. of measured, independent and observed [*I* > 2σ(*I*)] reflections	12421, 2826, 2658
*R*_int_	0.026
(sin θ/λ)_max_ (Å^−1^)	0.629

Refinement	
*R*[*F* ^2^ > 2σ(*F* ^2^)], *wR*(*F* ^2^), *S*	0.044, 0.129, 1.06
No. of reflections	2826
No. of parameters	233
No. of restraints	152
H-atom treatment	H-atom parameters constrained
Δρ_max_, Δρ_min_ (e Å^−3^)	0.24, −0.25

Computer programs: *CrysAlis PRO* (Rigaku OD, 2020 ▸), *SHELXT2018/2* (Sheldrick, 2015*a* ▸), *SHELXL2018/3* (Sheldrick, 2015*b* ▸) and *XP* (Siemens, 1994 ▸).

## full crystallographic data

### Crystal data

**Table:**

C_15_H_16_O_5_	*F*(000) = 584
*M**_r_* = 276.28	*D*_x_ = 1.344 Mg m^−^^3^
Monoclinic, *P*2_1_/*n*	Cu *K*α radiation, λ = 1.54184 Å
*a* = 11.4373 (2) Å	Cell parameters from 7342 reflections
*b* = 9.2045 (1) Å	θ = 4.2–76.0°
*c* = 13.9862 (2) Å	µ = 0.84 mm^−^^1^
β = 112.030 (2)°	*T* = 293 K
*V* = 1364.89 (4) Å^3^	Prism, colourless
*Z* = 4	0.30 × 0.25 × 0.20 mm

### Data collection

**Table:**

Rigaku Oxford Diffraction Xcalibur, Ruby diffractometer	*R*_int_ = 0.026
Radiation source: Enhance (Cu) X-ray Source	θ_max_ = 76.0°, θ_min_ = 5.9°
/ω scans	*h* = −13→14
Absorption correction: multi-scan (CrysAlisPro; Rigaku OD, 2020)	*k* = −11→11
*T*_min_ = 0.707, *T*_max_ = 1.000	*l* = −17→16
12421 measured reflections	3 standard reflections every 100 reflections
2826 independent reflections	intensity decay: 2.6%
2658 reflections with *I* > 2σ(*I*)	

### Refinement

**Table:**

Refinement on *F*^2^	Primary atom site location: dual
Least-squares matrix: full	Secondary atom site location: difference Fourier map
*R*[*F*^2^ > 2σ(*F*^2^)] = 0.044	Hydrogen site location: inferred from neighbouring sites
*wR*(*F*^2^) = 0.129	H-atom parameters constrained
*S* = 1.06	*w* = 1/[σ^2^(*F*_o_^2^) + (0.0784*P*)^2^ + 0.2588*P*] where *P* = (*F*_o_^2^ + 2*F*_c_^2^)/3
2826 reflections	(Δ/σ)_max_ = 0.001
233 parameters	Δρ_max_ = 0.24 e Å^−^^3^
152 restraints	Δρ_min_ = −0.25 e Å^−^^3^

### Special details

**Table:**

Geometry. All esds (except the esd in the dihedral angle between two l.s. planes) are estimated using the full covariance matrix. The cell esds are taken into account individually in the estimation of esds in distances, angles and torsion angles; correlations between esds in cell parameters are only used when they are defined by crystal symmetry. An approximate (isotropic) treatment of cell esds is used for estimating esds involving l.s. planes.
Refinement. All hydrogen atoms were placed in idealized positions and refined as riding. Methyl and hydroxyl H atoms were allowed to rotate but not to tip to best fit the experimental electron density. *U*_iso_(H) values were set to a multiple of *U*_eq_(C) with 1.5 for CH_3_ and OH, and 1.2 for C—H and CH_2_ units, respectively.

### Fractional atomic coordinates and isotropic or equivalent isotropic displacement parameters (Å^2^)

**Table:**

	*x*	*y*	*z*	*U*_iso_*/*U*_eq_	Occ. (<1)
O1	0.59277 (8)	0.31729 (9)	0.90699 (6)	0.0387 (2)	
O2	0.71917 (11)	0.16510 (11)	1.02027 (8)	0.0581 (3)	
O3	0.41699 (11)	0.86743 (10)	0.76809 (7)	0.0528 (3)	
O4	0.34926 (8)	0.63380 (10)	0.63957 (6)	0.0394 (2)	
O5	0.42264 (9)	0.35773 (10)	0.71482 (7)	0.0455 (3)	
H5A	0.361972	0.380320	0.663022	0.068*	
C1	0.68442 (12)	0.29021 (14)	1.00155 (10)	0.0408 (3)	
C2	0.73150 (12)	0.41184 (15)	1.07060 (10)	0.0435 (3)	
H2A	0.793928	0.396102	1.135298	0.052*	
C3	0.68748 (11)	0.54680 (14)	1.04379 (9)	0.0384 (3)	
H3A	0.717941	0.622790	1.090374	0.046*	
C4	0.59337 (11)	0.57447 (13)	0.94328 (9)	0.0332 (3)	
C5	0.54701 (12)	0.71408 (13)	0.90989 (9)	0.0372 (3)	
H5B	0.572267	0.792182	0.955223	0.045*	
C6	0.46380 (12)	0.73575 (13)	0.80966 (10)	0.0377 (3)	
C7	0.42467 (11)	0.61589 (13)	0.74204 (9)	0.0352 (3)	
C8	0.46321 (11)	0.47571 (13)	0.77651 (9)	0.0341 (3)	
C9	0.55012 (11)	0.45657 (12)	0.87717 (9)	0.0323 (3)	
C10	0.4522 (2)	0.98950 (16)	0.83649 (13)	0.0673 (5)	
H10B	0.415653	1.076267	0.799159	0.101*	
H10C	0.542443	0.998598	0.864752	0.101*	
H10D	0.422271	0.975472	0.891400	0.101*	
C11A	0.4264 (2)	0.6569 (3)	0.57834 (14)	0.0551 (5)	0.920 (3)
H11A	0.495799	0.588214	0.598800	0.066*	0.920 (3)
H11B	0.461255	0.754337	0.589740	0.066*	0.920 (3)
C12A	0.34670 (15)	0.63675 (18)	0.46744 (11)	0.0470 (4)	0.920 (3)
H12A	0.296120	0.554266	0.449743	0.056*	0.920 (3)
C13A	0.3414 (5)	0.7259 (3)	0.39174 (16)	0.0518 (6)	0.920 (3)
C14A	0.4154 (3)	0.8638 (3)	0.4076 (2)	0.0904 (8)	0.920 (3)
H14A	0.453102	0.870756	0.356890	0.136*	0.920 (3)
H14B	0.480385	0.863960	0.475330	0.136*	0.920 (3)
H14C	0.360338	0.945127	0.400707	0.136*	0.920 (3)
C15A	0.2580 (2)	0.6955 (5)	0.28248 (15)	0.0918 (9)	0.920 (3)
H15A	0.308261	0.688163	0.241029	0.138*	0.920 (3)
H15B	0.198125	0.772994	0.257010	0.138*	0.920 (3)
H15C	0.213889	0.605708	0.279173	0.138*	0.920 (3)
C11B	0.412 (3)	0.601 (2)	0.5635 (18)	0.047 (3)	0.080 (3)
H11C	0.500724	0.579784	0.600430	0.057*	0.080 (3)
H11D	0.372932	0.515912	0.522606	0.057*	0.080 (3)
C12B	0.3977 (19)	0.726 (2)	0.4962 (13)	0.058 (3)	0.080 (3)
H12B	0.438259	0.811199	0.527301	0.070*	0.080 (3)
C13B	0.333 (7)	0.732 (4)	0.3955 (17)	0.061 (4)	0.080 (3)
C14B	0.280 (2)	0.610 (3)	0.3204 (19)	0.068 (4)	0.080 (3)
H14D	0.327570	0.600656	0.276911	0.102*	0.080 (3)
H14E	0.193447	0.630179	0.278768	0.102*	0.080 (3)
H14F	0.285031	0.520783	0.357461	0.102*	0.080 (3)
C15B	0.340 (3)	0.874 (3)	0.345 (2)	0.083 (5)	0.080 (3)
H15D	0.261923	0.925182	0.328773	0.124*	0.080 (3)
H15E	0.355021	0.855862	0.283156	0.124*	0.080 (3)
H15F	0.407753	0.931049	0.391619	0.124*	0.080 (3)

### Atomic displacement parameters (Å^2^)

**Table:**

	*U*^11^	*U*^22^	*U*^33^	*U*^12^	*U*^13^	*U*^23^
O1	0.0444 (5)	0.0305 (4)	0.0309 (4)	0.0043 (3)	0.0022 (4)	−0.0006 (3)
O2	0.0673 (7)	0.0397 (5)	0.0455 (6)	0.0162 (5)	−0.0036 (5)	0.0023 (4)
O3	0.0732 (7)	0.0304 (5)	0.0369 (5)	0.0063 (4)	0.0001 (5)	0.0015 (4)
O4	0.0415 (5)	0.0409 (5)	0.0271 (4)	0.0033 (3)	0.0030 (3)	0.0017 (3)
O5	0.0540 (6)	0.0333 (5)	0.0322 (5)	0.0014 (4)	−0.0034 (4)	−0.0061 (3)
C1	0.0414 (6)	0.0386 (6)	0.0334 (6)	0.0074 (5)	0.0039 (5)	0.0036 (5)
C2	0.0403 (6)	0.0451 (7)	0.0312 (6)	0.0027 (5)	−0.0024 (5)	0.0007 (5)
C3	0.0385 (6)	0.0379 (6)	0.0306 (6)	−0.0043 (5)	0.0034 (5)	−0.0041 (5)
C4	0.0343 (6)	0.0331 (6)	0.0280 (5)	−0.0024 (4)	0.0067 (4)	−0.0009 (4)
C5	0.0443 (6)	0.0307 (6)	0.0307 (6)	−0.0030 (5)	0.0072 (5)	−0.0033 (4)
C6	0.0442 (6)	0.0295 (6)	0.0335 (6)	0.0008 (5)	0.0080 (5)	0.0017 (4)
C7	0.0370 (6)	0.0357 (6)	0.0265 (5)	0.0008 (5)	0.0044 (4)	0.0008 (4)
C8	0.0363 (6)	0.0325 (6)	0.0278 (6)	−0.0012 (4)	0.0054 (5)	−0.0038 (4)
C9	0.0347 (6)	0.0289 (5)	0.0290 (6)	0.0012 (4)	0.0072 (4)	0.0006 (4)
C10	0.1004 (14)	0.0298 (7)	0.0472 (8)	0.0073 (7)	−0.0003 (8)	−0.0003 (6)
C11A	0.0470 (9)	0.0785 (14)	0.0333 (9)	−0.0019 (10)	0.0077 (7)	0.0064 (9)
C12A	0.0507 (8)	0.0515 (8)	0.0348 (7)	−0.0030 (6)	0.0115 (6)	−0.0035 (6)
C13A	0.0497 (12)	0.0699 (11)	0.0369 (8)	0.0131 (8)	0.0174 (7)	0.0060 (8)
C14A	0.118 (2)	0.0772 (15)	0.0821 (16)	−0.0091 (14)	0.0441 (16)	0.0170 (12)
C15A	0.0702 (13)	0.170 (3)	0.0332 (9)	0.0054 (16)	0.0166 (9)	0.0043 (13)
C11B	0.043 (5)	0.060 (6)	0.039 (5)	0.002 (5)	0.016 (4)	−0.006 (5)
C12B	0.056 (4)	0.068 (5)	0.045 (4)	0.005 (4)	0.014 (4)	0.000 (4)
C13B	0.058 (5)	0.075 (5)	0.046 (5)	0.007 (5)	0.014 (5)	0.001 (5)
C14B	0.056 (8)	0.092 (9)	0.059 (8)	0.013 (8)	0.025 (7)	0.009 (8)
C15B	0.072 (9)	0.098 (9)	0.067 (9)	0.012 (8)	0.014 (8)	−0.008 (8)

### Geometric parameters (Å, º)

**Table:**

O1—C1	1.3675 (15)	C11A—H11A	0.9700
O1—C9	1.3794 (14)	C11A—H11B	0.9700
O2—C1	1.2143 (16)	C12A—C13A	1.323 (3)
O3—C6	1.3639 (14)	C12A—H12A	0.9300
O3—C10	1.4321 (17)	C13A—C15A	1.493 (3)
O4—C7	1.3776 (13)	C13A—C14A	1.495 (4)
O4—C11A	1.457 (2)	C14A—H14A	0.9600
O4—C11B	1.52 (3)	C14A—H14B	0.9600
O5—C8	1.3560 (14)	C14A—H14C	0.9600
O5—H5A	0.8200	C15A—H15A	0.9600
C1—C2	1.4442 (18)	C15A—H15B	0.9600
C2—C3	1.3400 (19)	C15A—H15C	0.9600
C2—H2A	0.9300	C11B—C12B	1.462 (17)
C3—C4	1.4368 (16)	C11B—H11C	0.9700
C3—H3A	0.9300	C11B—H11D	0.9700
C4—C9	1.3907 (16)	C12B—C13B	1.323 (18)
C4—C5	1.4017 (17)	C12B—H12B	0.9300
C5—C6	1.3819 (17)	C13B—C14B	1.50 (2)
C5—H5B	0.9300	C13B—C15B	1.50 (2)
C6—C7	1.4122 (17)	C14B—H14D	0.9600
C7—C8	1.3900 (16)	C14B—H14E	0.9600
C8—C9	1.3968 (15)	C14B—H14F	0.9600
C10—H10B	0.9600	C15B—H15D	0.9600
C10—H10C	0.9600	C15B—H15E	0.9600
C10—H10D	0.9600	C15B—H15F	0.9600
C11A—C12A	1.487 (2)		
			
C1—O1—C9	121.18 (10)	H11A—C11A—H11B	108.3
C6—O3—C10	116.44 (10)	C13A—C12A—C11A	125.7 (2)
C7—O4—C11A	110.36 (11)	C13A—C12A—H12A	117.2
C7—O4—C11B	115.4 (9)	C11A—C12A—H12A	117.2
C8—O5—H5A	109.5	C12A—C13A—C15A	121.5 (3)
O2—C1—O1	116.84 (12)	C12A—C13A—C14A	123.7 (2)
O2—C1—C2	125.54 (12)	C15A—C13A—C14A	114.8 (2)
O1—C1—C2	117.62 (11)	C13A—C14A—H14A	109.5
C3—C2—C1	121.65 (11)	C13A—C14A—H14B	109.5
C3—C2—H2A	119.2	H14A—C14A—H14B	109.5
C1—C2—H2A	119.2	C13A—C14A—H14C	109.5
C2—C3—C4	120.20 (11)	H14A—C14A—H14C	109.5
C2—C3—H3A	119.9	H14B—C14A—H14C	109.5
C4—C3—H3A	119.9	C13A—C15A—H15A	109.5
C9—C4—C5	119.87 (11)	C13A—C15A—H15B	109.5
C9—C4—C3	117.47 (11)	H15A—C15A—H15B	109.5
C5—C4—C3	122.64 (11)	C13A—C15A—H15C	109.5
C6—C5—C4	120.05 (11)	H15A—C15A—H15C	109.5
C6—C5—H5B	120.0	H15B—C15A—H15C	109.5
C4—C5—H5B	120.0	C12B—C11B—O4	108.9 (17)
O3—C6—C5	124.86 (11)	C12B—C11B—H11C	109.9
O3—C6—C7	115.70 (11)	O4—C11B—H11C	109.9
C5—C6—C7	119.42 (11)	C12B—C11B—H11D	109.9
O4—C7—C8	117.73 (10)	O4—C11B—H11D	109.9
O4—C7—C6	121.38 (10)	H11C—C11B—H11D	108.3
C8—C7—C6	120.89 (11)	C13B—C12B—C11B	127 (2)
O5—C8—C7	122.30 (10)	C13B—C12B—H12B	116.6
O5—C8—C9	118.99 (10)	C11B—C12B—H12B	116.6
C7—C8—C9	118.67 (10)	C12B—C13B—C14B	129 (2)
O1—C9—C4	121.79 (10)	C12B—C13B—C15B	115 (2)
O1—C9—C8	117.34 (10)	C14B—C13B—C15B	114 (2)
C4—C9—C8	120.85 (11)	C13B—C14B—H14D	109.5
O3—C10—H10B	109.5	C13B—C14B—H14E	109.5
O3—C10—H10C	109.5	H14D—C14B—H14E	109.5
H10B—C10—H10C	109.5	C13B—C14B—H14F	109.5
O3—C10—H10D	109.5	H14D—C14B—H14F	109.5
H10B—C10—H10D	109.5	H14E—C14B—H14F	109.5
H10C—C10—H10D	109.5	C13B—C15B—H15D	109.5
O4—C11A—C12A	108.98 (15)	C13B—C15B—H15E	109.5
O4—C11A—H11A	109.9	H15D—C15B—H15E	109.5
C12A—C11A—H11A	109.9	C13B—C15B—H15F	109.5
O4—C11A—H11B	109.9	H15D—C15B—H15F	109.5
C12A—C11A—H11B	109.9	H15E—C15B—H15F	109.5

### Hydrogen-bond geometry (Å, º)

**Table:**

*D*—H···*A*	*D*—H	H···*A*	*D*···*A*	*D*—H···*A*
O5—H5*A*···O4	0.82	2.35	2.7580 (13)	111
O5—H5*A*···O2^i^	0.82	2.09	2.8484 (13)	153
C11*B*—H11*D*···O2^i^	0.97	2.41	3.19 (3)	137
C12*A*—H12*A*···O2^i^	0.93	2.54	3.3468 (19)	145

Symmetry code: (i) *x*−1/2, −*y*+1/2, *z*−1/2.
